# Inflammatory blood markers in breast cancer: a narrative review from early detection to therapy response

**DOI:** 10.1097/MS9.0000000000003687

**Published:** 2025-08-05

**Authors:** Emmanuel Ifeanyi Obeagu

**Affiliations:** Department of Biomedical and Laboratory Science, Africa University, Mutare, Zimbabwe

**Keywords:** breast cancer, C-reactive protein, inflammatory markers, monocyte-to-lymphocyte ratio, neutrophil-to-lymphocyte ratio

## Abstract

Breast cancer remains the most frequently diagnosed malignancy among women worldwide, with early detection and accurate prognostication crucial for improving survival outcomes. While imaging and histopathological analyses are standard diagnostic tools, there is growing interest in cost-effective, minimally invasive biomarkers that can complement existing modalities – particularly in resource-limited settings. This narrative review explores the role of inflammatory blood markers, including neutrophil-to-lymphocyte ratio (NLR), platelet-to-lymphocyte ratio (PLR), monocyte-to-lymphocyte ratio (MLR), and systemic immune-inflammation index (SII), in breast cancer detection, prognosis, and monitoring of therapeutic response. These markers, derived from routine complete blood counts, reflect the systemic immune landscape and are increasingly associated with tumor progression, treatment resistance, and survival. We also highlight the limitations of these markers, particularly their non-specificity, and compare their utility with established diagnostic and molecular techniques. While not diagnostic alone, these inflammatory indices may serve as adjuncts in clinical decision-making and merit further validation in prospective studies.

## Introduction

Breast cancer is the most prevalent malignancy among women worldwide and remains a major cause of cancer-related mortality. Early detection and effective treatment strategies are critical in improving survival rates and quality of life for affected individuals. While conventional diagnostic methods such as mammography, ultrasound, and biopsy play essential roles in disease detection, there is a growing need for minimally invasive biomarkers that can aid in early diagnosis, risk stratification, and treatment monitoring. In this context, inflammatory blood markers have gained considerable attention due to their ability to reflect the systemic inflammatory response associated with cancer progression^[1–5]^. Inflammation is a well-established hallmark of breast cancer and plays a pivotal role in Breast tumor initiation, promotion, and metastasis. The tumor microenvironment is characterized by a complex interaction between cancer cells, immune cells, and inflammatory mediators that can either suppress or promote tumor growth. Systemic inflammatory markers such as C-reactive protein (CRP), neutrophil-to-lymphocyte ratio (NLR), monocyte-to-lymphocyte ratio (MLR), and platelet-to-lymphocyte ratio (PLR) have been identified as potential prognostic and predictive indicators in various malignancies, including breast cancer. These markers provide insights into the balance between pro-tumor inflammation and anti-tumor immune responses, making them valuable tools for assessing disease status^[6–12]^. Among the commonly studied inflammatory markers, CRP serves as a general indicator of systemic inflammation and has been associated with increased breast cancer risk, disease progression, and poorer survival outcomes. Similarly, elevated NLR has been linked to tumor aggressiveness, as higher neutrophil counts reflect a pro-inflammatory state, while reduced lymphocyte levels indicate weakened immune surveillance. MLR, another key marker, represents the balance between monocyte-driven inflammation and lymphocyte-mediated immune response, with higher values correlating with adverse clinical outcomes. PLR, which reflects the role of platelets in promoting tumor growth and metastasis, has also been shown to predict survival rates in breast cancer patients^[6–10]^.

**HIGHLIGHTS**

- Inflammatory blood markers such as CRP, NLR, and IL-6 offer promising tools for early breast cancer detection and prognosis.
- Chronic inflammation contributes significantly to tumor initiation, progression, and metastasis in breast cancer.
- Markers such as PLR and cytokines help predict patient responses to chemotherapy and immunotherapy.
- Integration of inflammatory markers into clinical practice may enhance personalized treatment and improve outcomes.
- Understanding the inflammation–cancer link reveals novel targets for both therapeutic intervention and disease monitoring.

Beyond their prognostic value, inflammatory markers have demonstrated significant potential in predicting responses to various treatment modalities. Studies have shown that pre-treatment NLR and MLR levels can influence chemotherapy effectiveness, with higher values associated with resistance to treatment and poorer outcomes. In the context of immunotherapy, markers such as MLR and systemic immune-inflammation index (SII) have been explored as potential predictors of response to immune checkpoint inhibitors. Additionally, inflammatory markers may play a role in monitoring toxicity and adverse effects related to cancer therapies, further highlighting their clinical relevance^[13–17]^. Additionally, inflammatory markers can be influenced by comorbid conditions such as infections, autoimmune disorders, and other malignancies, potentially limiting their specificity in breast cancer diagnostics. Recent advancements in artificial intelligence and machine learning have opened new avenues for improving the predictive accuracy of inflammatory markers in breast cancer. Computational models incorporating inflammatory markers with other clinical and molecular biomarkers could enhance risk prediction and treatment stratification. Moreover, large-scale prospective studies are essential to validate the clinical utility of these markers across diverse populations and breast cancer subtypes^[18–20]^. Several biochemical markers have been explored for their potential role in the early diagnosis of breast cancer, including CA15-3 and carcinoembryonic antigen (CEA), though with limited specificity and sensitivity[21]. Currently, estrogen receptor (ER), progesterone receptor (PR), and human epidermal growth factor receptor 2 (HER2) markers are widely used in routine clinical practice for breast cancer classification and therapeutic guidance[22]. Radiologic modalities such as mammography, ultrasound, and MRI remain integral to breast cancer detection and staging[23]. Assessing non-specific immune functions, such as natural killer (NK) cell activity, has also been explored as a potential immune-monitoring strategy in cancer patients. Building on this premise, our paper aims to highlight simple, accessible inflammatory blood markers that may support routine clinical evaluation of breast cancer[24]. In low-resource settings, the development and validation of low-cost, broadly applicable diagnostic markers are critical to improving breast cancer outcomes. It is important to note that NLR and PLR are non-specific markers and have been reported in numerous inflammatory and non-malignant conditions.

## Aim

The aim of this review is to explore the role of inflammatory blood markers in breast cancer, highlighting their significance in early detection, disease progression, prognosis, and response to therapy.

## Review methods

To provide a comprehensive and insightful analysis of the role of inflammatory blood markers in breast cancer, a structured yet flexible review methodology was adopted. The review was conducted using a narrative approach, aiming to synthesize existing evidence while allowing for the contextual integration of findings from various disciplines including oncology, immunology, and clinical pathology. A thorough literature search was carried out across multiple scientific databases, including PubMed, Scopus, Web of Science, and Google Scholar, focusing on articles published between 2010 and 2024. Keywords and phrases such as *“breast cancer,” “inflammatory markers,” “C-reactive protein,” “neutrophil-to-lymphocyte ratio,” “cytokines,” “tumor microenvironment,”* and *“therapy response”* were strategically used in different combinations to capture the breadth of relevant literature. Inclusion criteria emphasized peer-reviewed original research, systematic reviews, meta-analyses, and significant clinical trials that investigated the association of blood-based inflammatory markers with breast cancer detection, prognosis, and treatment outcomes. Only studies published in English and involving human subjects were considered. Each article was assessed for its scientific quality, relevance, and contribution to the understanding of the topic. Special attention was given to studies that included large patient cohorts, robust statistical analyses, and clear clinical implications. Where necessary, additional references from bibliographies of key articles were reviewed to ensure comprehensiveness. By integrating findings from these diverse sources, this review offers a narrative synthesis that reflects current understanding, highlights ongoing debates, and identifies gaps in knowledge regarding the diagnostic and prognostic potential of inflammatory blood markers in breast cancer.

## The role of inflammation in breast cancer

Long before a lump is discovered or a diagnosis is made, the groundwork for malignancy may be quietly laid through persistent, low-grade inflammation. This process is not a mere bystander in cancer development; it is an active participant, influencing the very origins, evolution, and behavior of tumors[2]. Inflammation is the body’s natural response to injury or infection. It’s the reason a cut turns red or a sore becomes swollen – immune cells rushing to repair damage. But what happens when this repair mechanism turns chronic, especially when there’s no clear injury to fix? This is where inflammation starts to go rogue, creating a microenvironment where cells can mutate, survive, and multiply unchecked[3]. In breast tissue, this scenario is particularly potent. Hormonal changes, environmental stressors, obesity, and genetic mutations can all contribute to a prolonged inflammatory state. As inflammatory cells infiltrate breast tissue, they release a cocktail of cytokines, chemokines, and growth factors. These substances, intended to heal, instead nourish abnormal cells, encourage their survival, and ultimately support the transformation from normal cells to malignant ones[4]. Among the major culprits are tumor-associated macrophages (TAMs), which, under the influence of chronic inflammation, shift from their usual role in immune defense to promoting tumor growth. These cells secrete vascular endothelial growth factor (VEGF), promoting the formation of new blood vessels that supply the tumor. At the same time, interleukin-6 (IL-6) and interleukin-8 (IL-8) create a favorable setting for cancer cells to resist apoptosis – the natural cell death that should occur when something goes wrong^[5,6]^. Inflammation doesn’t just start cancer – it sustains and strengthens it. As the tumor grows, it hijacks the inflammatory process further, recruiting more immune cells to support invasion and metastasis. The feedback loop continues: cancer fuels inflammation, and inflammation feeds the cancer[7]. But perhaps the most dangerous aspect of inflammation in breast cancer is its stealth. Many women with high levels of systemic inflammation have no visible symptoms. The inflammatory response smolders quietly, detectable only through subtle changes in blood markers such as C-reactive protein (CRP) or elevated neutrophil-to-lymphocyte ratios (NLR). These markers, once overlooked, are now gaining recognition as harbingers of risk and progression[8]. Even in treatment, inflammation plays a role. It can help predict how well a patient will respond to chemotherapy or indicate the likelihood of recurrence. In some cases, it may even contribute to resistance against therapy, making tumors harder to kill[9].

## Inflammatory blood markers in early detection of breast cancer

**Figure 1.**
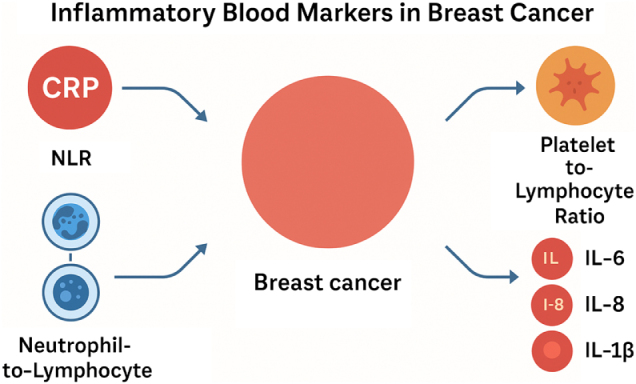
Inflammatory blood markers in breast cancer.

Early detection of breast cancer is critical in improving patient survival and reducing disease-related morbidity. Traditional diagnostic tools such as mammography, ultrasound, and biopsy are the gold standard for breast cancer detection, but they have limitations, including radiation exposure, high costs, and reduced sensitivity in patients with dense breast tissue. As a result, there is growing interest in identifying non-invasive, cost-effective biomarkers that can aid in early diagnosis. Inflammatory blood markers, including C-reactive protein (CRP), neutrophil-to-lymphocyte ratio (NLR), monocyte-to-lymphocyte ratio (MLR), and platelet-to-lymphocyte ratio (PLR), have emerged as promising candidates for early breast cancer detection due to their association with cancer-related inflammation and immune responses^[7–10]^. Systemic inflammation plays a significant role in carcinogenesis by promoting cellular proliferation, angiogenesis, and immune evasion. Elevated CRP levels, a well-established marker of systemic inflammation, have been linked to an increased risk of breast cancer development. High-sensitivity CRP (hs-CRP) assays have shown potential in detecting early-stage breast cancer, with studies reporting a correlation between elevated CRP levels and tumor presence. Similarly, an increased NLR has been associated with early tumorigenesis, as neutrophils contribute to tumor-promoting inflammation while lymphocytes play a crucial role in anti-tumor immunity. A high NLR at the time of diagnosis may indicate a shift toward a pro-tumor immune environment, making it a useful marker for identifying early-stage disease^[13–15]^. Several studies have demonstrated the utility of combining inflammatory markers with conventional screening methods to improve diagnostic accuracy. For instance, integrating CRP and NLR values with imaging techniques has been shown to enhance sensitivity and specificity in detecting breast cancer, particularly in patients with non-palpable tumors. Furthermore, MLR and PLR have been explored as potential screening tools, with elevated levels correlating with increased breast cancer risk. While inflammatory markers alone may not replace traditional diagnostic modalities, their incorporation into routine screening protocols may offer a non-invasive approach to identifying high-risk individuals and guiding further diagnostic investigations (Fig. 1). Future research should focus on establishing standardized cut-off values and validating these markers in large, diverse populations to optimize their use in breast cancer screening and early detection (Table 1)^[16–20]^.

**Table 1:** Inflammatory blood markers in breast cancer

Marker	Definition	Biological significance	Clinical relevance in breast cancer	Limitations
NLR (neutrophil-to-lymphocyte ratio)	Neutrophils/lymphocytes	Reflects balance between inflammation (neutrophils) and immune surveillance (lymphocytes)	Linked with poor prognosis, tumor grade, recurrence, and chemotherapy response	Non-specific; altered in infections, autoimmune diseases
PLR (platelet-to-lymphocyte ratio)	Platelets/lymphocytes	Indicates thrombocytosis and immune suppression	Associated with tumor aggressiveness and metastasis	Affected by thrombosis, inflammation, anemia
MLR (monocyte-to-lymphocyte ratio)	Monocytes/lymphocytes	Monocytes may reflect tumor-associated macrophage precursors	Correlated with lymph node involvement and survival	Also altered in systemic infections and autoimmune diseases
SII (systemic immune-inflammation index)	(Neutrophils × platelets)/lymphocytes	Integrates three immune-inflammatory pathways	Predictive of response to neoadjuvant chemotherapy	Requires standardization and population-specific cutoffs
Cytokines (e.g., IL-6, TNF-α, IL-1β)	Circulating pro-inflammatory proteins	Reflect systemic inflammation and tumor-immune crosstalk	May serve as prognostic indicators and therapeutic targets	Measurement variability, low disease specificity

## Prognostic significance of inflammatory markers in breast cancer

Inflammation plays a pivotal role in cancer progression, influencing tumor growth, metastasis, and response to therapy. In breast cancer, inflammatory blood markers such as C-reactive protein (CRP), neutrophil-to-lymphocyte ratio (NLR), monocyte-to-lymphocyte ratio (MLR), and platelet-to-lymphocyte ratio (PLR) have emerged as important prognostic indicators. These markers reflect the balance between pro-tumor inflammatory responses and anti-tumor immunity, providing valuable insights into disease progression and patient survival. Their ease of measurement and cost-effectiveness make them attractive candidates for routine clinical assessment in breast cancer prognostication^[10–12]^. Several studies have demonstrated a strong correlation between elevated inflammatory markers and poor clinical outcomes in breast cancer patients. High levels of CRP, a general marker of systemic inflammation, have been associated with increased tumor burden, lymph node involvement, and reduced overall survival. Similarly, an elevated NLR has been linked to aggressive tumor biology, as high neutrophil counts promote tumor progression by releasing pro-inflammatory cytokines, while reduced lymphocyte levels indicate impaired anti-tumor immune responses. A higher NLR has been consistently associated with worse disease-free survival (DFS) and overall survival (OS) across various breast cancer subtypes^[13–15]^. MLR and PLR have also been explored as prognostic markers in breast cancer. Increased MLR levels have been linked to enhanced monocyte-mediated tumor-promoting inflammation and poorer survival outcomes. Similarly, elevated PLR has been associated with increased platelet activation, which facilitates tumor angiogenesis and metastasis. Patients with higher PLR values tend to have a greater risk of recurrence and lower survival rates. Collectively, these inflammatory markers provide valuable prognostic information, helping clinicians stratify patients based on disease severity and guide treatment decisions. However, further research is needed to establish standardized cut-off values and validate their prognostic significance in large, prospective studies to enhance their clinical applicability in breast cancer management^[16–18]^.

## Inflammatory blood markers and therapy response in breast cancer

The response to therapy in breast cancer is influenced by multiple factors, including tumor biology, genetic mutations, and the patient’s immune and inflammatory status^[21,22]^. Inflammatory blood markers such as C-reactive protein (CRP), neutrophil-to-lymphocyte ratio (NLR), monocyte-to-lymphocyte ratio (MLR), and platelet-to-lymphocyte ratio (PLR) have gained attention as potential indicators of treatment response^[23,24]^. These markers reflect the systemic inflammatory environment and the balance between immune activation and suppression, providing valuable insights into how a patient might respond to chemotherapy, targeted therapy, and immunotherapy. Several studies have demonstrated that elevated NLR and PLR are associated with poor responses to chemotherapy in breast cancer patients. A high NLR indicates a predominance of neutrophils, which contribute to tumor-promoting inflammation, and a reduced lymphocyte count, signifying impaired anti-tumor immunity. Patients with higher NLR values before chemotherapy initiation often exhibit lower pathological complete response (pCR) rates and increased chemotherapy resistance. Similarly, an elevated PLR has been linked to enhanced platelet-mediated tumor progression and worse treatment outcomes. Conversely, a lower MLR, reflecting a more favorable immune profile, has been associated with better chemotherapy response and improved survival^[25–27]^. In the context of immunotherapy and targeted therapies, inflammatory markers may also serve as predictors of treatment efficacy. Immunotherapy, particularly immune checkpoint inhibitors (ICIs), relies on the activation of the host immune system to target and destroy cancer cells. High levels of inflammation, reflected by elevated NLR and CRP, have been associated with reduced responses to ICIs due to an immunosuppressive tumor microenvironment^[27–29]^. Additionally, inflammatory markers have been explored in patients receiving targeted therapies such as HER2 inhibitors and CDK4/6 inhibitors, where lower NLR and MLR values have been linked to improved treatment response^[30,31]^. As research advances, integrating inflammatory markers into routine clinical practice may help personalize treatment strategies, optimize patient selection for therapies, and improve overall breast cancer outcomes. However, further prospective studies are needed to validate their role in predicting therapy response and refine their clinical application^[32–34]^.

## Conclusion

Inflammatory blood markers represent a promising avenue for improving breast cancer management, from early detection to therapy response assessment. Their non-invasive nature, affordability, and ability to provide real-time insights into disease progression make them valuable tools in oncology. While further research is needed to refine their clinical application, integrating inflammatory markers into routine breast cancer care has the potential to enhance diagnostic precision, prognostication, and personalized treatment strategies.

### Study limitations

This review, while offering comprehensive insights into the clinical utility of inflammatory blood markers in breast cancer, has several limitations that should be acknowledged:

1. Narrative nature of the review

This work is a narrative review, not a systematic review or meta-analysis. As such, the selection of literature may carry inherent bias and is not exhaustive. Quantitative synthesis of the data was not performed, and effect sizes or pooled outcomes were not calculated.

2. Non-specificity of inflammatory markers

The inflammatory indices discussed – such as NLR, PLR, MLR, and SII – are not specific to breast cancer. They are altered in a variety of pathological and physiological states, including infections, autoimmune diseases (e.g., systemic lupus erythematosus), psychiatric disorders, and other malignancies. This limits their diagnostic precision when used in isolation.

3. Lack of standardized cut-offs

The absence of universally accepted cut-off values for these markers complicates their application in routine clinical settings. Most studies use population-specific thresholds, which may not be generalizable.

4. Influence of confounding factors

Hematological markers can be influenced by age, medications (e.g., steroids, chemotherapy), comorbidities, and individual immune responses, which were not controlled for in many of the studies cited.

5. Need for prospective validation

Many findings regarding inflammatory markers in breast cancer are based on retrospective or single-center studies. Prospective, multicenter, and population-specific studies are needed to validate their utility as prognostic or predictive tools.

6. Limited integration with molecular and imaging data

Although this review attempts to bridge inflammatory markers with existing diagnostic tools, it does not provide a robust analysis of how these markers correlate with molecular subtypes, radiologic findings, or liquid biopsy data in an integrated clinical workflow.

## Data Availability

Not applicable as this a narrative review.

## Research registration unique identifying number (UIN)

Not applicable as this a narrative review.
